# High-intensity light therapy for post-interventional complications and pain after minimally invasive spinal procedures: a case series

**DOI:** 10.1007/s10103-026-04815-6

**Published:** 2026-02-18

**Authors:** Lance Lorenzo Babcock, Billy K Huh, Saba Javed

**Affiliations:** 1 McGovern Medical School, The University of Texas Health Science Center at Houston, Houston, TX USA; 2 Department of Pain Medicine, The University of Texas MD Anderson Cancer Center, Houston, TX USA

**Keywords:** High intensity laser therapy, Minimally invasive spinal intervention, Pain management, Case series

## Abstract

High Intensity Laser Therapy (HILT) has improved postoperative outcomes by promoting faster recovery and analgesia. However, there is little evidence of its effectiveness in the context of recovery after minimally invasive spinal procedures. This case series analyzes the postoperative outcomes after minimally invasive spinal surgery by measuring pain, complications, and tissue oxygenation. Two cohorts of 16 patients, who were spinal surgery outpatients at MD Anderson Cancer Center, were separated into a HILT group and a control (no HILT) group. In the HILT group, pain levels and tissue oxygenation were assessed before and after each of two HILT sessions. The postoperative complications for each group were compared at each of the two follow-up visits. Tissue oxygenation levels were significantly higher after each HILT application at each visit, and pain was significantly reduced after each HILT application compared to before treatment. There was no significant difference observed in the postoperative complications between the control group and the HILT group. These results suggest that the use of HILT in the minimally invasive spinal intervention postoperative setting improves patient outcomes.

**Supplementary Information:**

The online version contains supplementary material available at 10.1007/s10103-026-04815-6.

## Introduction

High-Intensity Laser Therapy (HILT) is a non-invasive tool to promote healing and post-surgical recovery. HILT uses a pulsed high-intensity light with a wavelength of 600–1300 nm, which is not easily absorbed by the body’s chromophores, allowing the energy to penetrate more deeply into the tissue [1]. Biostimulation by laser therapy promotes fibroblast proliferation, deposition of collagen, and extracellular matrix synthesis [2]. HILT is used to increase blood flow, alleviate pain, improve wound healing, and mitigate post-surgical complications [3–5].

The proposed mechanisms by which HILT therapy works are multifold. Laser energy can deeply penetrate the skin, and it may impact several cellular mechanisms. The Cytochrome C Oxidase enzymes in mitochondria increase Adenosine Triphosphate (ATP) production when they absorb light in the 580–700 nm range [6, 7]. This range overlaps with the possible range of High Intensity Laser Therapy treatment. Along with ATP production, nitric oxide (NO) can also be produced [8]. NO is a vasodilatory substance that can increase microcirculation blood flow. Endogenous transforming growth factor-beta1 can also be indirectly photoactivated, contributing to wound healing [9]. Membrane transporters can also be modulated by absorbing light energy, resulting in the exchange of calcium, protons, sodium, and potassium to/from the cell, which might provide analgesic responses and modulate inflammatory responses [10].

HILT has many potential therapeutic applications and improves postoperative outcomes. It has effectively helped manage conditions such as musculoskeletal disorders, like osteoarthritis and tendinopathies [11, 12]. Chronic pain conditions like low back pain, temporomandibular joint disorders, and sports-related injuries have also been shown to improve after HILT therapy [13–15]. In the postoperative setting, HILT has provided significant analgesic effects and enhanced tissue oxygenation, promoting faster recovery [16, 17]. Promoting wound healing with non-invasive procedures like HILT reduces the risk of postoperative complications, like infections. However, it has not yet been demonstrated if HILT therapy might provide the same benefits for recovery after minimally invasive spinal procedures for pain management.

This case series seeks to contribute to the growing body of evidence regarding the impact of High-Intensity Laser Therapy (HILT) on postoperative outcomes, specifically pain, complications, and tissue oxygenation. We hypothesize that HILT therapy significantly enhances tissue oxygenation, alleviates postoperative pain, and reduces the incidence of complications following minimally invasive spinal intervention.

## Case description

### Patient selection

This case series is based on data obtained through an institutional review board-approved, retrospective analysis of outpatients who underwent minimally invasive spinal surgery at MD Anderson Cancer Center (MDACC). Although these procedures were performed in an operative setting, they are considered minimally invasive spinal interventions rather than open spinal or spinal cord surgery. These patients were chronic pain medicine patients with a past or remote history of cancer, now cancer-free, and followed in the pain clinic for ongoing management of chronic pain. A waiver of informed consent was granted for each case. Data were collected from procedures performed between August 2018 and June 2023. Two cohorts, each consisting of 16 patients, were matched based on age and type of minimally invasive spinal intervention—Spinal Cord Stimulator implant/explant, Intrathecal Pain Pump implant/exchange, or Dorsal Root Ganglion implant—and were divided into a HILT treatment group and a control group (no HILT therapy). Patient ages ranged from 49 to 74 years. In the HILT group, pain levels and tissue oxygenation were assessed both before and after each of the two HILT sessions. Postoperative complications, including wound dehiscence and erythema, were evaluated in both groups during standard postoperative follow-up visits: day 5 (corresponding to HILT session 1) and day 10 (HILT session 2 and staple removal).

### HILT treatment

High-Intensity Laser Therapy (HILT) was administered using a device provided by Curewave Lasers (TX, USA), delivering power outputs between 1 and 44 watts and operating within a wavelength range of 660 to 1300 nm, as described in our previous study [18]. This system is capable of penetrating soft tissue to a depth of 5 to 15 cm. The laser operates in a pulsed mode to reduce thermal buildup and minimize the risk of heat-related tissue damage [19]. Each treatment session lasted approximately 90 s and was performed by trained rehabilitation staff using a standardized protocol. The laser was applied directly over the surgical site and surrounding soft tissues in a sweeping, circular motion to ensure even energy distribution. Patients received two treatment sessions: one on postoperative day 5 and another on day 10, coinciding with standard follow-up care. No adverse effects related to HILT were observed or reported during the study period.

### Pain reporting

Pain severity was quantified using the Visual Analogue Scale (VAS), a clinically validated interval scale ranging from 0 to 10, where 0 represents no pain and 10 denotes the worst possible pain. This scale is widely recognized for its reliability in assessing self-reported pain levels [20–22]. Patients in the HILT therapy treatment group were asked to report their pain scores at three key time points: prior to the first laser therapy session, on postoperative day 5 (after the first HILT session), and on postoperative day 10 (after the second HILT session and staple removal). These measurements allowed for the evaluation of pain progression and the immediate effects of HILT therapy on postoperative discomfort.

### Tissue oxygenation measurement

Tissue oxygen supply was measured with a Tissue Oxygen Sensory, or T-stat 2.0 (Spectros Medical Devices, TX, USA), which is a fiber optic white light sensor. Skeletal muscle oxygenation was continually measured before then after HILT therapy at the incision sites for each patient (midline, paramidline, flank, lumbar, or abdomen).

### Data analysis

Statistical analysis was performed in GraphPad Prism 10. Fisher’s exact test was used to analyze postoperative complications between control and HILT therapy groups. Paired T-test was used to analyze Tissue Oxygenation at visit 1 and visit 2. Friedman test and Dunn’s multiple comparisons test were used to analyze postoperative pain ratings at the three time points.

## Results

**Fig. 1:**
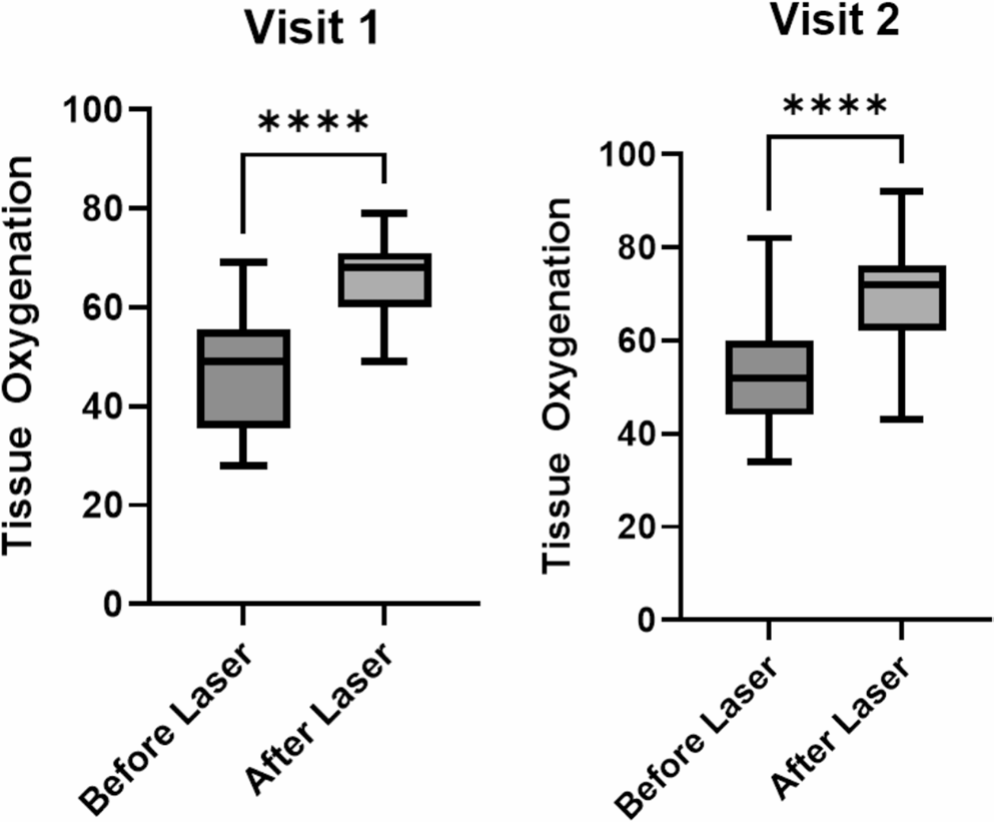
Comparison of tissue oxygenation in the HILT therapy cohort before HILT therapy (Before Laser) and after HILT therapy (After Laser). Visit 1 was at post-operation day 5 and Visit 2 was at post-operation day 10. ****P-value < 0.0001. *P*-value is from Paired T-test

We measured oxygenation at the surgical sites before and after HILT treatment to evaluate the change in tissue oxygenation with HILT treatment. Tissue oxygenation was significantly higher after HILT therapy than before HILT therapy at both visit 1 (day 5 post-op) and visit 2 (day 10 post-op) (Fig. 1). At visit 1, the mean of differences of before HILT therapy compared to after therapy was 19.34%. At visit 2, the mean of differences of before HILT therapy compared to after HILT therapy was 17.72%.

**Fig. 2:**
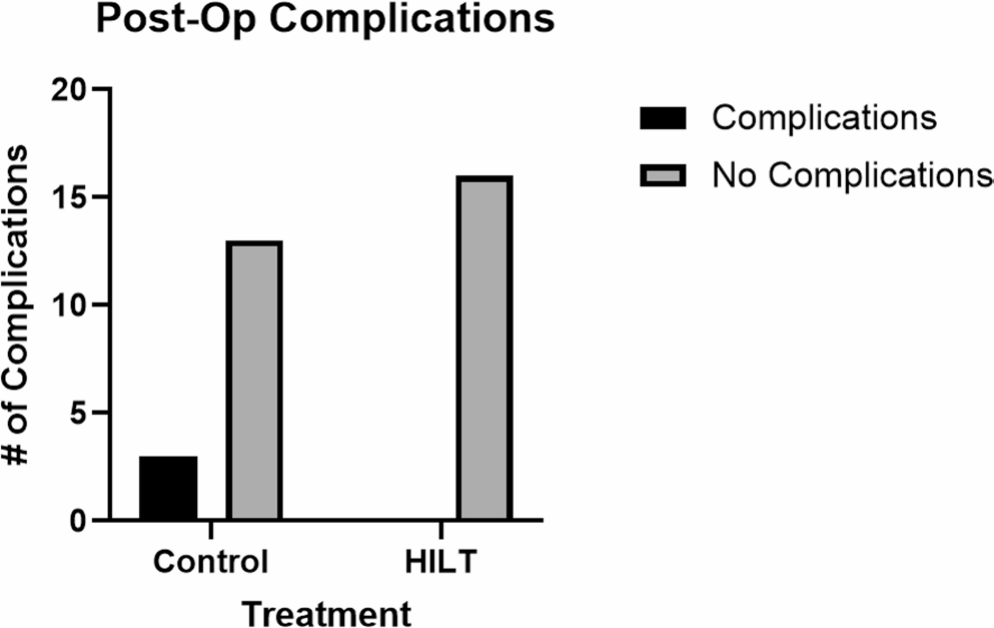
Comparison of post-operation complications between Controls and HILT therapy groups. *P*-value is from Fisher’s exact test

All enrolled patients were monitored for post-operation complications, like wound dehiscence and erythema, throughout the duration of the study. Post-operation complications were increased in the control group (3/16), but not in the HILT therapy group (0/16). This difference was not statistically significant (P-value = 0.2258) (Fig. 2).

**Fig. 3:**
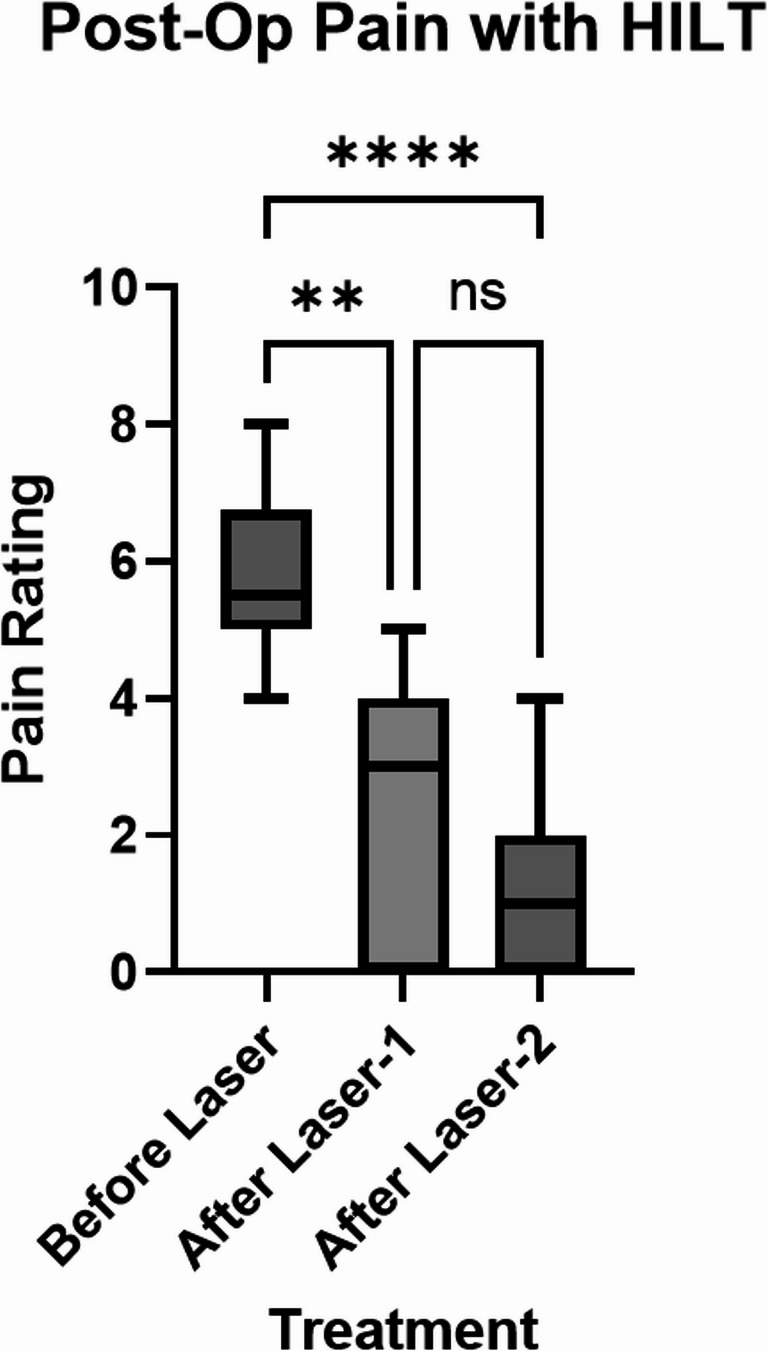
Comparison of subjective pain rating by each patient in the HILT therapy group before treatment (Before Laser), by post-operation day 5 (After Laser-1), and by post-operation day 10 (After Laser-2). **P-value = 0.0012, ****P-value < 0.0001, ns: not significant. *P*-value is from Friedman test and Dunn’s multiple comparisons test

The patients assigned to the HILT-therapy condition reported their pain ratings at three time points. Compared to before laser therapy (Before Laser), subjective pain was significantly decreased by post-op day 5 (After Laser 1) and further decreased by post-op day 10 (After Laser 2) (Fig. 3). There was no significant difference in the average pain rating between the first treatment and the second treatment. The greatest difference in pain rating was observed between before HILT treatment to after the second treatment. The average pain rating was 5.7 before HILT, 2.3 after the first treatment, and 1.1 after the second treatment.

## Discussion

Maximizing the benefits and minimizing the risks associated with spinal implant surgery is critical to continue advancing the growing field of neuromodulation. Our case series explored how the implementation of High Intensity Laser Therapy (HILT) impacted the course of recovery in postoperative minimally invasive spinal intervention patients. HILT is a non-invasive tool that has been shown to both shorten healing time and alleviate pain [3–5]. Our results demonstrated that the use of HILT therapy in our cohort of minimally invasive spinal procedure patients significantly increased tissue oxygenation and decreased postoperative pain, but there was no statistically significant difference in postoperative complications between the control and HILT treatment groups. While not demonstrated in this paper, another benefit of HILT is that each session can take less than 10 min [2]. This allows for the efficient treatment of many patients in a single day. Taken together, the implementation of HILT into the postoperative care plan can be an efficient and effective way to improve patient outcomes.

Previous studies demonstrated increases in tissue oxygenation with HILT therapy, so we measured this parameter in our patients after minimally invasive spinal surgery [16, 17]. Both visits demonstrated a significant increase in tissue oxygenation after using HILT therapy. The average increase in tissue oxygenation at the measured sites was 19.34% at visit 1 and 17.72% at visit 2. However, we did not measure how long this increase in tissue oxygenation lasted. While there appeared to be no significant difference in the “Before” therapy conditions from visit 1 to visit 2, the improvement of oxygenation by the end of the visit can promote healing at the operation site. As shown by Kösters et al. (2017), improved tissue oxygenation to a wound site is correlated with faster healing times and improved outcomes (appearance, less scarring, lower risk of complications). These long-term benefits are likely due to improved blood flow to the treated sites. Subsequent experiments measuring how much time this increased tissue oxygenation is sustained will aid in designing optimal therapeutic timing between HILT treatments.

Previous studies have shown that HILT treatment is related to a shorter wound healing time compared to controls without HILT therapy. In Thabet et al. (2018), the caesarean section wound size, in women with delayed healing, was significantly smaller in the HILT-treated group compared to the sham laser-treated group by 6 weeks of HILT therapy. Shorter healing times reduce the amount of time the wound is vulnerable to complications. Importantly, no wound complications, skin erythema, or adverse local effects were observed in any patient in this study following HILT administration. We observed 3 of the 20 subjects in the control group and 0 of the 20 subjects in the HILT group develop a postoperative complication. However, this difference was not statistically significant. This lack of observed significant difference may stem from the many precautions already in place in the medical field to prevent postoperative complications. The risk of complications is low, < 2% [23]. This indicates that large sample sizes may be needed to observe a significant difference between the groups.

Pain at the sites of incision is common after surgery. We employed HILT to assess whether this therapy will decrease pain after minimally invasive spinal interventions, similar to that observed in previous studies using HILT after other operations [4]. In this study, we found that patients reported significantly less pain after each HILT treatment compared to before treatment. Taken together with the tissue oxygenation data, one potential explanation of this reduced pain is that increased blood flow to the wound site decreases the accumulation of inflammatory cytokines. Another possible explanation is that the light modifies the activity of nociceptive cells by modulating the transport of molecules like calcium. While there are many proposed mechanisms, HILT therapy will need more thorough investigations with experimental designs to conclude definite molecular mechanisms in vivo [24].

## Limitations

Some limitations of HILT are that it is more expensive than Low Intensity Laser Therapy and requires the patient to return to the clinic for treatment [25]. Implementation of HILT in the postoperative setting may encounter some loss to follow up, and therefore, less effective treatment of the surgical site. This issue can be addressed by making HILT more widely available.

Hypothetically, it is reasonable to assume that treating a wound to promote faster healing with light will only reduce complications. Faster healing decreases the amount of time the surgical site is vulnerable to complications. However, the statistical power of this study was not high enough to reveal a statistically significant difference when compared to the non-HILT treatment control group. More than 16 subjects in each group are needed to adequately conclude if there may be a benefit provided by HILT in terms of postoperative complications. Overall, A major limitation of this study is the very small sample size of 16 patients per condition, which restricts the generalizability of our findings. Larger controlled studies are necessary to draw definitive conclusions regarding the therapeutic benefits of HILT in this patient population.

While the results display a significant decrease in reported postoperative pain after each laser therapy compared to before laser therapy, it is necessary to compare these results to a group not receiving HILT to draw conclusions. Without this control group, it is not possible to tell how much of an effect HILT had on reducing the pain rating. Visit 1 took place 5 days after the surgery and visit 2 was 10 days after the surgery. With enough time, postoperative pain usually decreases without treatment. Subsequent experiments are needed to compare a minimally invasive spinal surgery post-operation group receiving HILT to a control group receiving conventional pain management.

## Conclusion

In this case series, we compared HILT-therapy to non-HILT-therapy in patients after minimally invasive spinal intervention to assess pain, tissue oxygenation, and postoperative complications. Overall, these results indicate that the use of HILT in the minimally invasive spinal procedure postoperative setting can improve patient outcomes. Larger studies using HILT in the postoperative setting are necessary to further support its widespread implementation.

## Supplementary Information

Below is the link to the electronic supplementary material.

Supplementary Material 1

## Data Availability

Data is provided within the supplementary information files.
